# Pilot study on the use of cold atmospheric plasma for preservation of bread

**DOI:** 10.1038/s41598-022-26701-1

**Published:** 2022-12-20

**Authors:** Agnieszka Starek-Wójcicka, Renata Różyło, Iwona Niedźwiedź, Michał Kwiatkowski, Piotr Terebun, Magdalena Polak-Berecka, Joanna Pawłat

**Affiliations:** 1 Department of Biological Bases of Food and Feed Technologies, University of Life Sciences in Lublin, 28 Głęboka St., 20-612 Lublin, Poland; 2 Department of Food Engineering and Machines, University of Life Sciences in Lublin, 28 Głęboka St., 20-612 Lublin, Poland; 3 Department of Microbiology, Biotechnology and Human Nutrition, University of Life Sciences in Lublin, 8 Skromna St., 20-704 Lublin, Poland; 4 Institiute of Electrical Engineering and Electro Technologies, Lublin University of Technology, 38a Nadbystrzycka St., 20-618 Lublin, Poland

**Keywords:** Applied physics, Biophysics, Microbiology, Engineering, Materials science

## Abstract

Cold atmospheric plasma (CAP) is used as an emerging technology for food preservation. In this study, CAP treatment has been applied to bakery products for the first time. The aim of the work was to investigate the effect of the use of CAP on the amount of microorganisms during bread storage. Basic physicochemical properties and bread texture were determined during storage for 0, 3, and 6 days. The study material included gluten-free and mixed wheat-rye bread treated with CAP for 2 and 10 min. The results showed that no mesophilic bacteria or fungi were found after ten minutes of the bread exposure to CAP. In addition, only 2-min non-thermal sterilization resulted in complete inhibition of yeast and mould growth in the gluten-free and wheat-rye bread. A decrease in the microbial growth in the bread was noted; however, a simultaneous decrease in the moisture content of the bread was observed. After the application of plasma for 2 or 10 min, both the gluten-free and mixed wheat-rye bread was characterized by reduced humidity, which also resulted in a significant increase in the hardness and a slight increase in the springiness of the bread. The use of CAP in storage of bread is promising; nevertheless, it is necessary to further study the effect of this treatment in bread with improvers, especially with hydrocolloids and fibers.

## Introduction

The emerging cold plasma (CP) technology is increasingly often being used in the food industry, e.g. as an alternative tool for food decontamination and extension of the shelf life^1–4^. Cold plasma is generated as a result of non-thermal ionization of gas into free electrons, ions, reactive atomic and molecular forms, and ultraviolet (UV) radiation. It can be used to alter the surface of solid and liquid food products and has many advantages over traditional heat treatment. It seems that cold plasma has a limited effect on sensory and color properties at lower power and processing time. At higher intensity and a longer cold plasma treatment time, changes in the structure of carbohydrates, i.e. cross-linking and glycosylation, changes in the secondary structure of proteins, and oxidation of lipids may occur^5^. Therefore, research on the selection of parameters for various food products is necessary.

Some attempts have been made using different food products, but bread has never been treated with CP before storage. Bread is one of the most basic foods in the world; hence, scientists are highly interested in improving its quality. Improvement of technological parameters should be targeted at extension of its shelf life. Many scientists focus on delaying the staling of bread by various additives. The staleness of bread is caused by the retrogradation of amylopectin and the redistribution of water between different polymers. Non-starch proteins and polysaccharides also play a role in this process^6^. In addition to staleness, bakery products lose quality through the growth of mold and other microorganisms^7^.

All these changes occurring during storage have an impact on the increase in the amount of waste worldwide^6^; therefore, more attention should be paid to extending the shelf life of bread. Studies have been conducted to inhibit mold growth with the use of modified atmosphere packaging involving volatile mustard essential oil. This technology is based on the use of CO_2_ balanced with N_2_ and residual O_2_^8^. Other studies have tested an active packaging material, i.e. a multilayer film containing essential oil of star anise and a layer of thyol coating with insect repellent and antimicrobial properties. The film effectively inhibited the growth of microorganisms on the bread surface^9^. Other authors have indicated that ethanol can be used to slow down or avoid fungal spoilage. Techniques using this substance were effective in delaying the growth of *C. sitophila* and *H. burtoni*a^10^.

More efficient methods are constantly being sought in terms of extending the shelf life of bread. Our innovative proposal is the use of cold plasma. It is an emerging technology in non-thermal food preservation. Cold plasma is an electrically energized matter consisting of highly reactive species, which contains charged molecules and gas with minute particles in the form of negative and positive ions, photon electrons, and free radicals at room temperature. An increase in plasma-based food treatment used for inactivation of food-borne pathogens has been observed in recent years. The current study shows the activity of plasma agents on the microbial population, the surface sanitation of raw products in food processing, and future novelties in the food technology^11–13^. There are reports on the application of non-thermal plasma for beverages such as fresh juices and wine^13–20^.

Cold plasma treatment has been studied as a non-thermal method of inhibiting *Penicillium italicum* and improving the storage of mandarins. These results demonstrated the potential of using cold plasma treatment as a postharvest technology to preserve mandarins and increase the total phenolic content and antioxidant effects of mandarin peels^21^. Other authors have studied the effects of a microbial sanitizing system that integrates washing with antibacterial solutions and atmospheric dielectric barrier discharge cold plasma (ADCP) treatment on preservation of mandarins. The results of the study demonstrated the potential of this treatment to improve the storability of mandarins in plastic packaging by inhibiting the growth of *P. digitatum* on fruit while minimizing changes in fruit quality during storage^22^. Cold plasma treatment has been applied to inhibit food-borne pathogens and extend the shelf life of rocket leaves^23^ and fresh lettuce^24^. In the latter case, the authors confirmed the bacteriostatic effect against the growth of *E. coli*, and demonstrated the potential to improve the microbiological safety of vegetables without loss of physicochemical or sensory properties^25^. In other studies, authors determined^26,27^ the efficacy of Atmospheric Cold Plasma in reduction of potential microbiological contaminants of wheat grains. The aim of other studies was to determine the effect of the use of cold plasma on the functional properties of cereal raw materials, including wheat flour^28–30^ or rice starch^31^.

The bakery industry generates large amounts of food wastes due to the relatively short expiry time of bakery products, especially those without any artificial chemical agents added. To the best of our knowledge, the impact of cold atmospheric pressure plasma on bread has not been well investigated so far. Hence, this is a pioneering study in the field of bakery products and the first attempt to determine the effect of the use of cold plasma on microbes present in stored bread. In addition, the basic physicochemical properties, morphological structure, and texture of the bread were determined during storage after the plasma treatment. Mixed wheat-rye bread and gluten-free bread with different physical and sensory properties were selected to confirm the potential application of plasma-based technologies in bakery industry on a larger scale.

## Materials and methods

### Material

The gluten-free bread was prepared from maize and rice flours. The maize flour (Melvit, Warsaw, Poland) was characterized by the following nutrient contents: 83.8 ± 3.1% of carbohydrates, 7.1 ± 0.2% of protein, 0.49 ± 0.03% of ash, and 2.1 ± 0.1% of fat. The rice flour (Melvit, Warsaw, Poland) contained 78.9 ± 2.7% of carbohydrates, 7.2 ± 0.3% of protein, 0.31 ± 0.01% of ash, and 0.8 ± 0.03% of fat.

The basic raw materials for the preparation of the mixed wheat-rye bread were white wheat flour (Polskie Młyny, Warsaw, Poland) with 72.4 ± 3.1% of carbohydrates, 11.5 ± 0.5% of proteins, 0.69 ± 0.01% of ash, and 2.3 ± 0.1% of fat. In addition to the basic ingredients, dry instant yeast (Instaferm, Lallemand Iberia) and salt were added to the recipe.

### Bread baking procedure

The laboratory process of gluten-free bread baking was performed according to Zdybel et al.^32^ and Ziemichód et al.^33^ using a single-phase method. The recipe consisted of equal shares of rice and corn flour (50:50%). The flour and all other ingredients, i.e. yeast (1%) and salt (2%), were mixed (5 min) with water (100%), and the dough was transferred to molds for fermentation (30 °C, 40 min) in a fermentation cabinet (Sadkiewicz Instruments, Bydgoszcz, Poland). The bread was baked (230 °C, 40 min) in the laboratory oven (Sadkiewicz Instruments, Bydgoszcz, Poland).

Mixed wheat-rye bread baking was performed according to a straight-dough method described by Różyło et al.^34^ and Zdybel et al.^32^. The recipe consisted of equal shares of wheat and rye flour (50:50%), yeast (1%), salt (2%), and water (55%). All ingredients were mixed (5 min) and fermented (30 °C, 60 min) with 1-min kneading at half time. After the fermentation process, the dough was molded, proved (30 °C, 60 min), and baked (230 °C, 30 min). The baking was done in triplicate. After baking, the bread was cooled for 1 h, treated with cold atmospheric plasma, and stored.

### Plasma treatment

**Figure 1:**
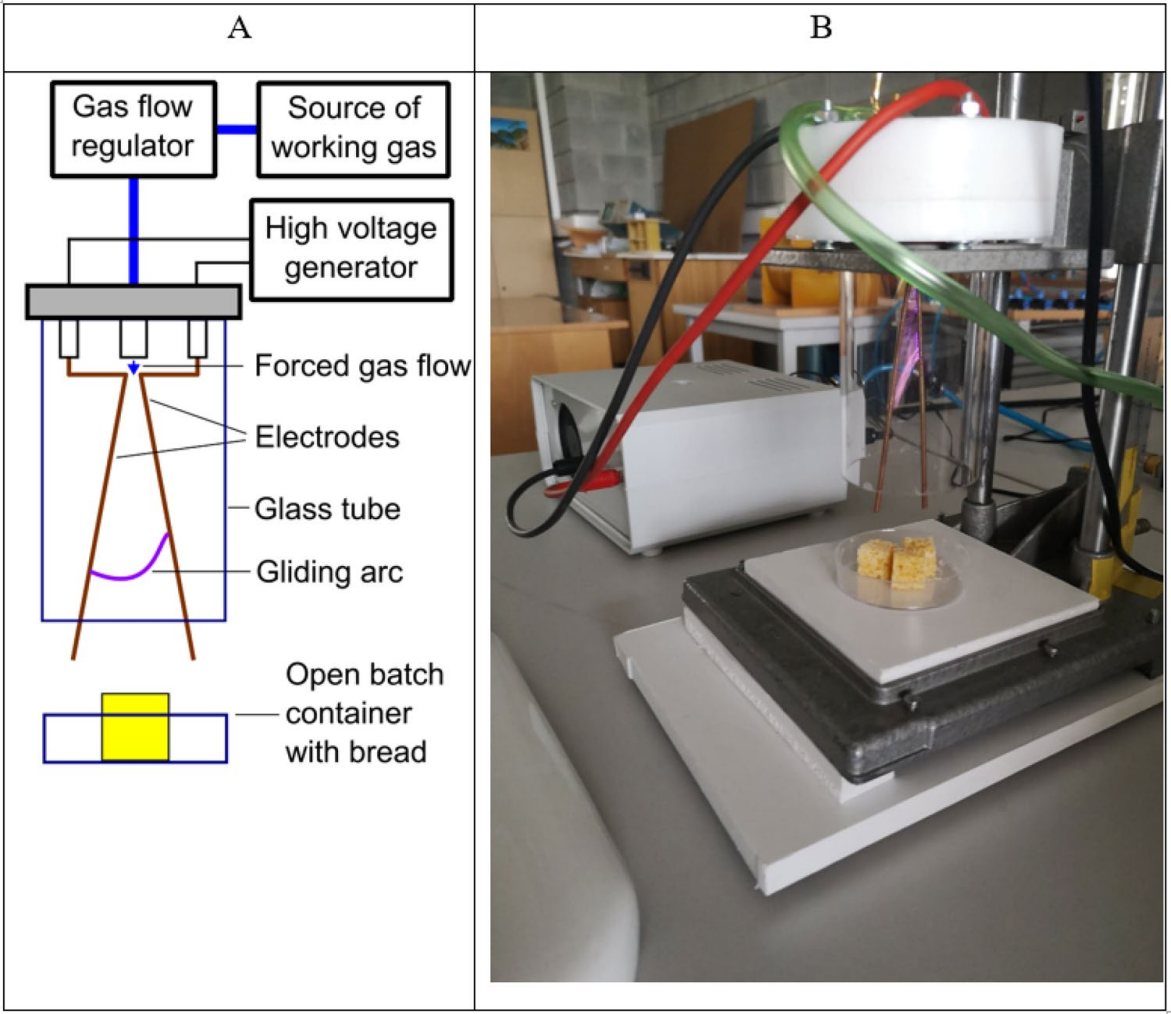
Experimental set-up (**A** scheme, **B** photograph).

A two-electrode gliding arc discharge (GAD) reactor operating at atmospheric pressure was applied for the treatment of the bread samples. The electrodes were made of profiled copper wires, 1.5 mm in diameter and 100 mm long, with an angle of 12 degrees between them. The discharge appeared at the shortest distance between the electrodes (3 mm), and then moved along the electrodes as a result of the forced gas flow. The flow rate of the nitrogen gas (purity 6.0, Linde Gas Poland) was adjusted by a gas flow controller (Automation Factory “ROTAMETR”, Gliwice, Poland) to 440 L/h. In order to prevent the gas from spreading out, a glass tube with a diameter of 50 mm was used (Fig. 1A). The power supply was based on an electronic high voltage transformer with the main frequency of 50 Hz. The converter of the power supply provided a series of irregular high voltage micro-pulses with a frequency of 20 kHz during 10 ms, thanks to which the transformer worked in the cut-off state for another 10 ms. During the treatment, the reactor was powered by an RMS (root-mean-square) voltage of 680 V (3.7 kV peak voltage) and apparent power of 40 VA. Bread samples were cut into 15 × 15 × 15 mm cubes and placed in the shallow open-batch glass container located under the GAD reactor’s gas outlet at the vertical distance of 1 cm between the sample surface and electrode’s tip. The photograph and scheme of the set up are presented in Fig. 1. After 2-min and 10-min plasma exposure, the temperature of the samples was measured using a DT-847U temperature meter with Type K thermocouple (Yu Ching Technology Co., Ltd., Taipei, Taiwan).

The results of previous studies carried out with the use of time-integrated optical emission spectroscopy confirmed the non-equilibrium nature of the discharge, where the vibrational temperature (from 3500 to 5500 K depending on the position of the arc) was much higher than the rotational temperature (from 1200 to 2200 K)^35^. Due to the after-glow effect, the average gas temperature at the treatment distance was relatively low and did not exceed 38 °C. When operating in an ambient air environment, the reactor allows both oxygen and nitrogen compounds to be generated relatively quickly, with a much higher concentration of nitrogen compounds^36^.

In the case of the plasma treatment of bread, the maximum concentrations of selected species achieved after the 10-min treatment were as follows: NO—390 ppm, NO_2_—20 ppm, CO—68 ppm, and O_3_—0.04 ppm. Nitrogen species and carbon monoxide were measured using MX6 iBrid (Industrial Scientific Corporation. Pittsburgh, PA USA) and the ozone concentration was monitored with Eco Sensors A-21ZX (Eco Sensors, Newark, CA, USA).

### Basic physicochemical measurements of bread

All physicochemical analyses of bread were performed after 0, 3, and 6 days of storage. The protein content was determined using a Kjeltec apparatus (TM8400, Foss, Victoria, AUS) and ASN 3100 software. The distillation was performed on an automated Kjeltec Auto analyzer (Tecator). In the Kjeldahl procedure^37^, after digestion in concentrated sulfuric acid, the total organic nitrogen is converted to ammonium sulfate. Ammonia is formed and distilled into a boric acid solution in alkaline conditions. The borate anions formed were titrated with standardized hydrochloric acid, on the basis of which the nitrogen content was calculated. The protein content was calculated from the nitrogen content using the conversion factor N × 5.7.

Total fat was determined using a Soxtec apparatus (Foss Analytical Solutions Pty. Ltd., Victoria, AUS). The sample was placed in a porous thimble and extracted in petroleum ether for 60 min within individual extraction tins (AN 310 Soxtec TM 8000 of Crude Fat using Extraction System). Then, it was placed in a laboratory drier for 30 min at 105 °C to further remove (vaporize) any residual solvent. The variation in the sample weight before and after the extraction was used to calculate the crude fat content expressed as a percentage of dry weight. The moisture content of the bread was determined with the ICC analysis of protein content method (1996).

All physicochemical analyses were performed in triplicate.

### Textural analysis of bread

The parameters of the bread crumb texture were determined using the TPA test, with double compression of the bread sample at a distance of 50% and a speed of 1 mm s^−1^ (ZWICK Z020/TN2S)^32^. The hardness and springiness^38^ were evaluated during the measurements. The texture measurements of the central part of bread crumb samples were performed in 6 replicates.

### Microbiological analysis

The control and cold atmospheric plasma-treated gluten-free bread and wheat-rye bread samples were analyzed to determine the total number of mesophilic bacteria and the total yeast and mould count. All microbiological analyses of the tested materials were carried out on storage days 0, 3, and 6 (18 to 20 °C, 75% humidity). Bread samples were prepared by shaking in saline for 15 min. on INFORS HT Minitron (INFORS AG CH-4103, Bottmingen, Switzerland) at 30 °C and 200 rpm. Ten-fold serial dilutions were then prepared aseptically via transferring 1 ml of each solution sample obtained into a 9-ml tube with saline. The evaluation of the microbiological purity of the tested materials was performed using the pour-plate method. Suitable decimal dilutions of the samples were plated, overlaid with sterilized nutrient agar medium (BTL, Łódź, Poland), and incubated for 72 h at 30 °C for total mesophilic bacteria. Sabouraud agar (BTL, Łódź, Poland) with chloramphenicol and incubation for 5 days at 25 °C were used for determination of total yeast and mold counts. After incubation, colonies were counted and the number of viable cells were determined as a mean of log colony forming units (cfu) per ml of sample ± standard deviation^39^.

### Microscopic observations

The control and plasma-treated bread samples were observed with an optical microscope KEYENCE VHX 950F (Japan) coupled with a digital camera to evaluate the effect of the plasma treatment on bread morphology.

### Statistical analyses

The statistical analysis of the results was carried out using Statistica 12.0 (α = 0.05). Analysis of variance (ANOVA) was performed, and Tukey’s test was used to compare the mean values.

## Results and discussion

### Physicochemical parameters of bread after cold atmospheric plasma treatment

After the plasma exposure, the temperature of the samples increased by maximum 14 °C compared to the control. Interestingly, due to the differences in the morphological structure of the bulk material, differences in heat accumulation were observed between the gluten-free and mixed wheat-rye bread (Table 1).

**Table 1:** Temperature of bread samples before and after plasma treatment.

Plasma treatment time/bread type	0 min	2 min	10 min
Gluten-free	23 °C	26.6 °C	32 °C
Mixed wheat-rye	23 °C	26.9 °C	37 °C

**Figure 2:**
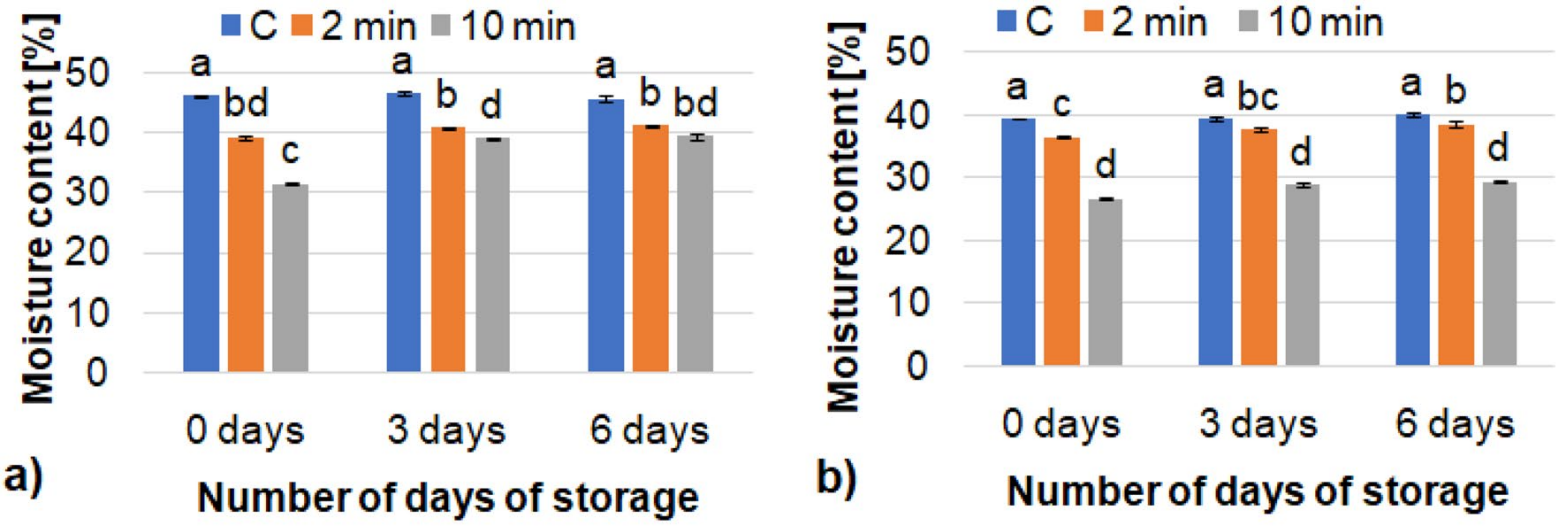
Moisture content of bread, (**a**) gluten-free bread, (**b**) mixed wheat-rye bread; mean values in the same figure marked with different letters are significantly (α = 0.05) different.

Subsequently, it was shown that the treatment of both the gluten-free and mixed wheat-rye bread with CAP gradually reduced the moisture content (Fig. 2) in these loaves. The increase in the cold plasma exposure time from 2 to 10 min resulted in lower moisture content of the bread. There were no significant differences in the moisture content of the bread during storage for 0, 3, and 6 days. This was probably due to the fact that the bread loaves were stored in closed foil bags. There is no research on the use of CAP to preserve bread, but other researchers^40^ used plasma to extend the storage time of fresh pasta. In their research, they noted that plasma allowed rapid removal of moisture and thus improved the shelf life of the pasta. The authors explain that the rapid migration of moisture molecules occurs due to the synergism between the structure driving force and the drying driving force caused by cold plasma. In meat samples (dry cured beef product)^41^, a decrease in moisture content was caused by CAP due to evaporation of water from the surface of the sample.

Our research showed that the application of plasma for 2 or 10 min induced changes in the texture of both the gluten-free and mixed wheat-rye bread (Table 2). The longer the plasma application time, the greater the increase in bread hardness. The use of plasma for 2 min resulted in a significant increase in bread hardness after three days of storage, but only by about 12%, while the use of plasma for 10 min increased the hardness of the gluten-free bread by 28%. Much greater changes in hardness were observed in the case of the mixed wheat-rye bread. However, this bread was characterized by significantly lower hardness than the gluten-free bread. Besides hardness, the springiness of both the gluten-free bread and the mixed wheat-rye bread increased as well. It should also be mentioned that, after 6 days of storage of the control bread, the bread was covered with mold and it was not possible to perform texture analysis and compare its results with the plasma-treated breads. There are no available results on bread texture parameters because no such tests have been performed yet. Studies on the influence of plasma treatment on the functional properties of food are still scarce. Other studies on the functional properties of wheat flour demonstrated that plasma treatment increased the flour hydration properties of wheat flour. Rapid visco-analyzer results showed an increase in pasting and the final viscosities of wheat flour. The authors explained that the decrease in both endothermic enthalpies and crystallinity was attributed to the depolymerization of starch and plasma-induced changes^28^.

**Table 2:** Changes in the texture of bread treated with cold atmospheric plasma.

Type of bread	Type of treatment	0 days		3 days		6 days	
		Hardness (N(	Springiness (–)	Hardness (N)	Springiness (–)	Hardness (N)	Springiness (–)
Gluten-free bread	Control	29.67 ± 1.55 cd	1.48 ± 0.16b	31.1 ± 0.7c	1.42 ± 0.18b	Mould growth	Mould growth
	CAP- 2 min	34.47 ± 1.07b	1.59 ± 0.05ab	35.0 ± 0.88b	1.45 ± 0.04b	35.86 ± 1.59b	1.42 ± 0.03b
	CAP-10 min	37.47 ± 1.37a	1.68 ± 0.07a	39.9 ± 1.7a	1.64 ± 0.12a	39.30 ± 0.61a	1.57 ± 0.12a
Mixed wheat-rye bread	Control	6.41 ± 0.51f.	0.82 ± 0.01d	8.2 ± 1.7e	0.82 ± 0.02d	Mould growth	Mould growth
	CAP– 2 min	7.73 ± 0.57e	0.86 ± 0.01c	13.8 ± 1.8d	0.84 ± 0.04 cd	13.161.27c	0.79 ± 0.01d
	CAP– 10 min	23.12 ± 1.39	0.78 ± 0.03d	33.3 ± 1.9b	0.87 ± 0.02c	33.38 ± 0.79b	0.88 ± 0.06c

*Mean values in the same column marked with different letters are significantly (α = 0.05) different.

**Figure 3:**
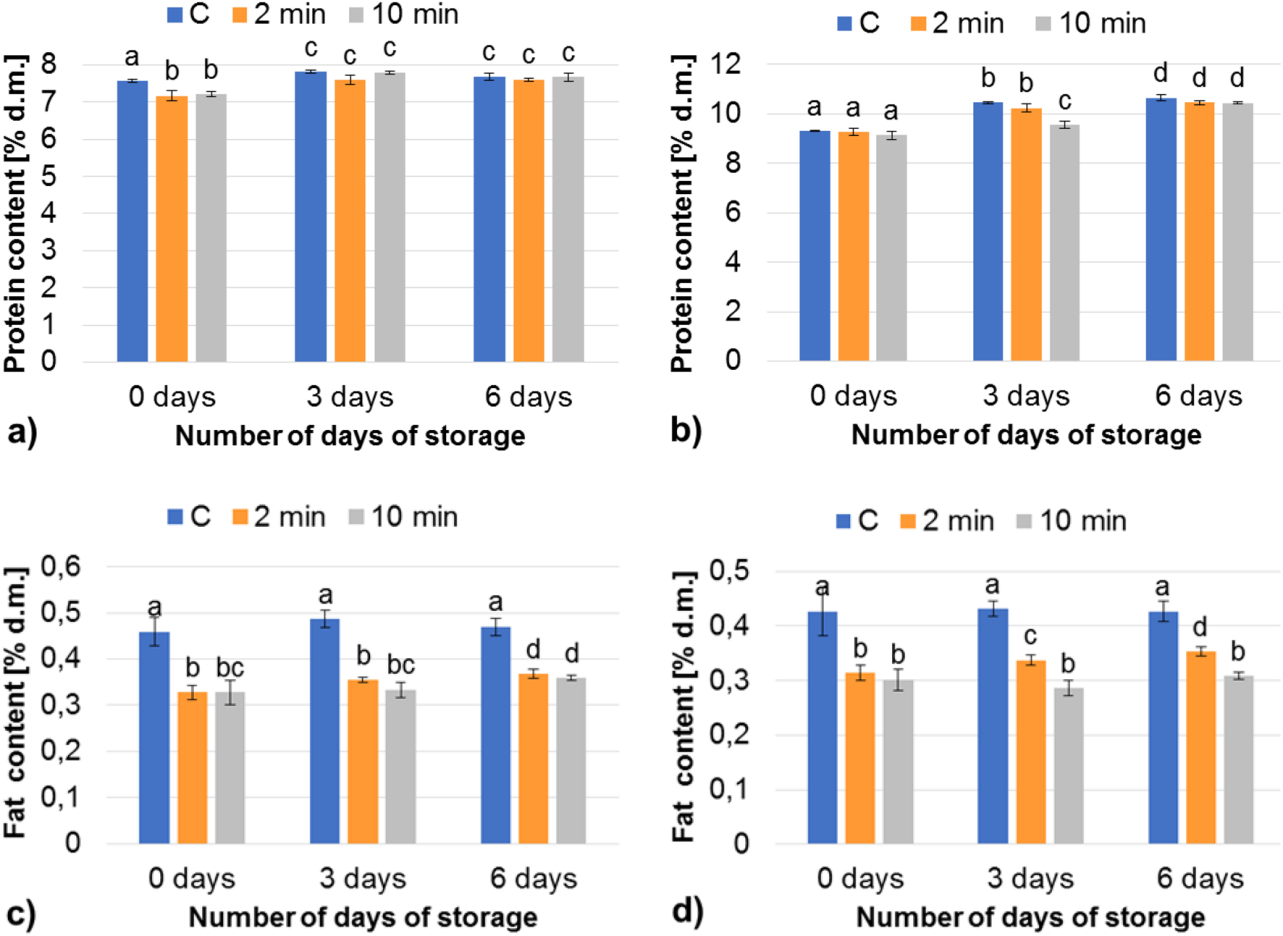
Protein (**a**, **b**) and fat (**c**, **d**) content of bread, (**a**, **c**) gluten-free bread, (**b**, **d**) mixed wheat-rye bread; mean values in the same figure marked with different letters are significantly (α = 0.05) different.

Interesting results were also obtained for the protein and fat (Fig. 3) content. In most cases, no significant differences in protein content were detected after the application of cold plasma to both the gluten-free and wheat-rye bread. In other work presented by Bahrami^1^, total proteins in wheat flour were also not significantly influenced by cold plasma treatment, although there was a trend towards higher molecular weight fractions, which indicated protein oxidation, and the treated flour did produce thicker dough. Other authors^30^ suggested that CAP treatments of wheat flour could change the protein structure, which appeared as differences in the rheological properties. In that study, the CAP treatment time, applied voltage, and their interaction, irrespective of the flour type, exerted a significant effect on the secondary structure of flour proteins. In other studies^28^, plasma treatment increased the wheat flour hydration properties. As explained by the authors, cold plasma can generate such reactive species as molecular oxygen and ozone, which are also the most common and universal oxidizing agent used for wheat flour conditioning. Other researchers^42^ demonstrated that plasma exposure did not have any adverse effect on rice storage proteins although the authors mention earlier studies reporting that oxidation of proteins and disulfide bond formation may be caused by ozonation.

In our study, the CAP treatment of bread slightly reduced the fat (Fig. 3c,d) content of the bread after the 2-min treatment (by around 28% for both baked products). The extension of the processing time to 10 min did not significantly affect this parameter, compared to the 2-min plasma exposure. In a study conducted by Bahrami et al.^1^, cold plasma treatment of wheat flour did not affect the concentration of total non-starch lipids and glycolipids. This treatment, however, reduced total free fatty acids and phospholipids. Oxidation markers (hydroperoxide value and head space n-hexanal) increased with the treatment time and the voltage of discharge, which confirmed the acceleration of lipid oxidation.

### Microbiological analysis of bread after cold atmospheric plasma treatment

**Figure 4:**
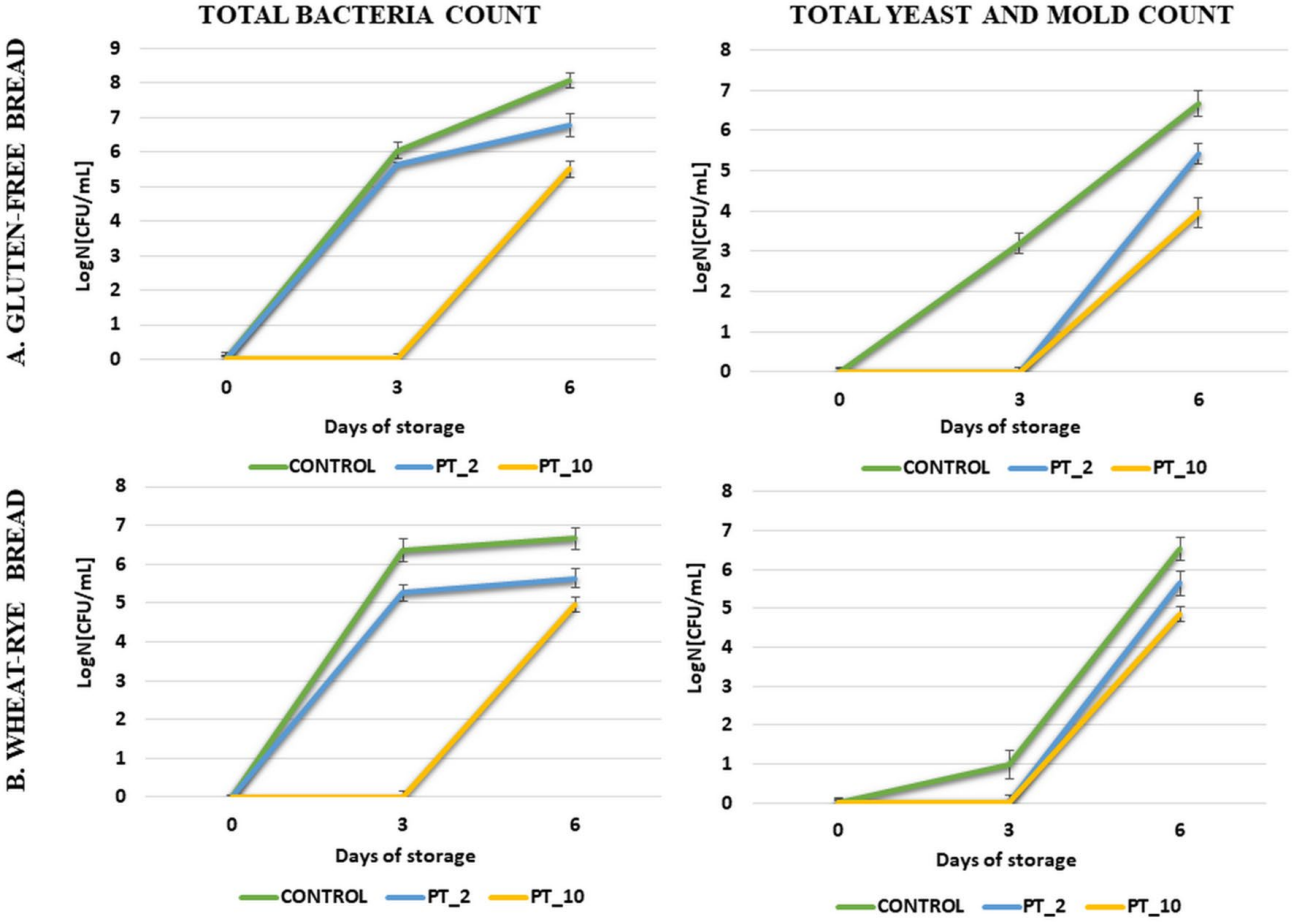
Effect of CAP treatment for 0, 2, and 10 min and storage at 20 °C for 0–6 days on the total mesophilic bacterial count and total yeast and mold growth in gluten-free bread and wheat-rye bread. Vertical bars depict standard error (PT_2—2 min plasma treatment, PT_10—10 min plasma treatment).

Microbiological contamination is a serious problem in the food industry, which scientists are attempting to solve by a constant search for new methods of food preservation. The constant growth of the human population makes the problem of hunger more apparent. It is therefore extremely important to extend the shelf life of food products to reduce food waste. In this work, the quantitative analysis of microbiological contamination of gluten-free and wheat-rye bread samples subjected to cold atmospheric plasma (CAP) treatment and then stored for 0, 3, and 6 days was performed. As shown in Fig. 4, microbiological contamination at a detectable level was recorded in the control samples of each type of bread after 3 days of storage. The total number of mesophilic bacteria in the gluten-free and wheat-rye bread was 6.04 ± 0.23 log_10_ and 6.36 ± 0.31 log_10_ CFU/ml, respectively, and the total number of yeasts and moulds was 3.2 ± 0.24 and 1 ± 0.37 log_10_ CFU/ml, respectively. However, after the 10-min CAP exposure of all bread samples stored for the same time, no mesophilic bacteria or fungi were found. In addition, only the 2-min non-thermal sterilization resulted in complete inhibition of yeast and mould growth in the gluten-free and wheat-rye bread. Moreover, the total count of mesophilic bacteria was lower by 1.1 log in the gluten-free bread, compared to the control. These results suggest that the 10-min exposure of bread samples to cold plasma may effectively extend the shelf life, while the 2-min exposure has a positive effect on limiting the development of unfavorable microflora. In the case of bread samples stored for 6 days, the development of microflora was noted in each variant. Nevertheless, reduced microbial growth was still observed in the cold plasma-exposed samples, compared to the control bread. However, the degree of growth inhibition depended on the duration of the exposure. The 2-min exposure of the samples to CAP resulted in a decrease in the growth of the total number of bacteria by 1.3 log and 1.02 log in the gluten-free and wheat-rye bread, respectively. The extension of the process time to 10 min contributed to a reduction in the number of bacteria by 2.57 log in the gluten-free bread sample and by 1.71 log in the wheat-rye bread sample, compared to the controls. In turn, the highest inhibition of the growth of undesirable yeast and mould cells was obtained after the 10-min exposure of the gluten-free bread samples, where the total number of viable cells was by 2.71 log lower than in the control sample. No studies have yet used cold plasma to extend the shelf life of bread, but CAP treatment of a dry beef product was shown to reduce the total mesophilic aerobic bacteria and yeast–mold counts^41^. In addition, the beef samples were inoculated with *Staphylococcus aureus* and *Listeria monocytogenes* and subjected to CAP. The study showed that CAP can be used to lower *S. aureus and L. monocytogenes* counts as well. Other studies^43^ were carried out to assess the bactericidal mechanism of CAP against *Escherichia coli* (*E. coli*). The results showed that the morphology of *E. coli* cells was altered by the charged particles and active ingredients produced by CAP. The cell wall and membrane of *E. coli* ruptured, the contents of the cells leaked, the cells lost their ability to reproduce and self-replicate, and the function of cell metabolism was directly affected and led to inactivation of the bacteria.

### Morphology of plasma-treated samples

**Figure 5:**
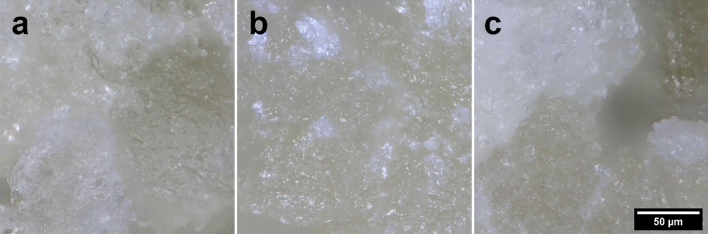
Images of gluten-free bread samples taken with an optical microscope: (**a**) control; (**b**) 2-min treatment; (**c**) − 10-min treatment.

**Figure 6:**
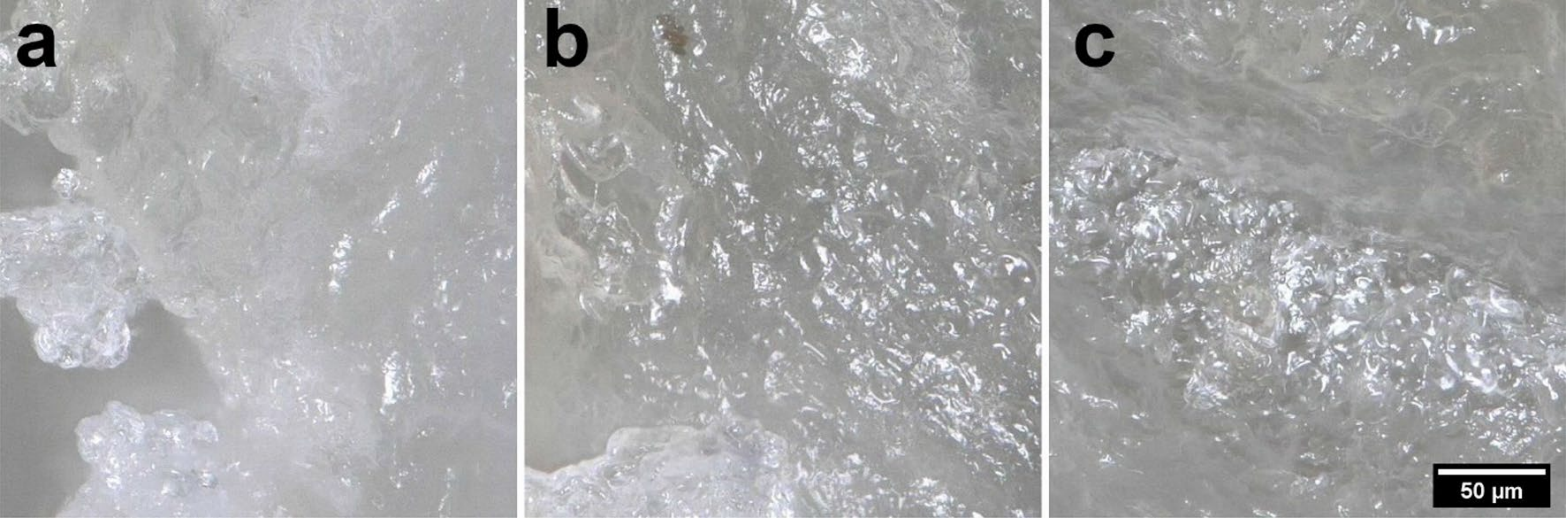
Images of wheat-rye bread samples taken with an optical microscope: (**a**) control; (**b**) 2-min treatment; (**c**) − 10-min treatment.

The microscopic observations revealed that the plasma treatment slightly enhanced porosity and thinned the pore walls because of the drying process. Water molecules that were present in the walls of the pores gradually evaporated and were replaced by air. However, even the 10-min plasma treatment did not cause distinctive morphological deterioration of the structure, as can be observed in Figs. 5 and 6. After the plasma treatment, light-reflective zones were observed, which may have been associated with further development of a crystalline network of amylose and amylopectin, which primarily tends to develop during the baking process^44,45^.

## Conclusion

Compared with other technologies, plasma has been widely regarded as a green, safe, and promising technology. Our research showed that both gluten-free and mixed wheat-rye bread, after the application of plasma for 2 or 10 min, was characterized by reduced humidity, which also induced changes in the texture of the bread during storage. The hardness and springiness of the bread increased. These parameters increased with the extension of the plasma-treatment time from 2 to 10 min. After the application of plasma, the protein content increased in the fresh mass of bread, while there were no significant changes in the dry matter. The use of the cold plasma technology in the process of storing bread is promising, as it contributes to a decrease in the growth of microorganisms. No mesophilic bacteria or fungi were found after the 10-min CAP exposure of all bread samples stored for the same time. In addition, only the 2-min of non-thermal sterilization resulted in complete inhibition of yeast and mould growth in the gluten-free and wheat-rye bread. In this study, we tested natural breads without technological enhancers. Other results may be obtained in analyses of loaves with hydrocolloids or other improvers; therefore, it is necessary to conduct further research.

## Data Availability

The datasets used and/or analyzed during the current study available from the corresponding authors on reasonable request.
